# Landscape-Scale Epidemiological Dynamics of SARS-CoV-2 in White-Tailed Deer

**DOI:** 10.1155/2024/7589509

**Published:** 2024-02-10

**Authors:** Joshua Hewitt, Grete Wilson-Henjum, Derek T. Collins, Timothy J. Linder, Julianna B. Lenoch, Jonathon D. Heale, Christopher A. Quintanal, Robert Pleszewski, Dillon S. McBride, Andrew S. Bowman, Jeffrey C. Chandler, Susan A. Shriner, Sarah N. Bevins, Dennis J. Kohler, Richard B. Chipman, Allen L. Gosser, David L. Bergman, Thomas J. DeLiberto, Kim M. Pepin

**Affiliations:** ^1^Department of Wildland Resources, Utah State University, Logan, UT, USA; ^2^National Wildlife Disease Program, United States Department of Agriculture, Fort Collins, CO, USA; ^3^Wildlife Services, United States Department of Agriculture, Fort Collins, CO, USA; ^4^Wildlife Disease Diagnostic Laboratory, United States Department of Agriculture, Fort Collins, CO, USA; ^5^Veterinary Preventive Medicine, The Ohio State University College of Veterinary Medicine, Columbus, OH, USA; ^6^National Wildlife Research Center, United States Department of Agriculture, Fort Collins, CO, USA

## Abstract

Understanding pathogen emergence in new host species is fundamental for developing prevention and response plans for human and animal health. We leveraged a large-scale surveillance dataset coordinated by United States Department of Agriculture, Animal and Plant Health Inspection Service and State Natural Resources Agencies to quantify the outbreak dynamics of SARS-CoV-2 in North American white-tailed deer (*Odocoileus virginianus*; WTD) throughout its range in the United States. Local epidemics in WTD were well approximated by a single-outbreak peak followed by fade out. Outbreaks peaked early in the northeast and mid-Atlantic. Local effective reproduction ratios of SARS-CoV-2 were between 1 and 2.5. Ten percent of variability in peak prevalence was explained by human infection pressure. This, together with the similar peak infection prevalence times across many counties and single-peak outbreak dynamics followed by fade out, suggest that widespread transmission via human-to-deer spillover may have been an important driver of the patterns and persistence. We provide a framework for inferring population-level epidemiological processes through joint analysis of many sparsely observed local outbreaks (landscape-scale surveillance data) and linking epidemiological parameters to ecological risk factors. The framework combines mechanistic and statistical models that can identify and track local outbreaks in long-term infection surveillance monitoring data.

## 1. Introduction

Starting in 2020, SARS-CoV-2 was found in white-tailed deer (WTD) [1, 2]. By 2021, there was evidence of regional transmission in WTD through a combination of ongoing deer-to-deer and human-to-deer transmission [2–5]. Early reports of SARS-CoV-2 in WTD were from surveillance in local areas—a single state, province, or region—during a 3- to 4-month window [1, 2, 4, 6]. Experimental infection studies corroborated that WTD are susceptible to SARS-CoV-2 infection, capable of shedding and deer-to-deer transmission, and able to form persisting neutralizing antibodies [7–9]. Endemic transmission of SARS-CoV-2 in WTD could position these populations as reservoir hosts, posing risk for variant persistence [4, 10], evolution of new variants [6, 11], and spillback into human populations [6, 11, 12]. Phylogenetic studies provide evidence that animal–human transmission and viral evolution routinely occurs in pandemics [13–15]. The potential for ongoing zoonotic outbreaks highlights the need to understand drivers of zoonotic pathogens establishing and persisting in new species to inform science-based One Health decisions, improve risk assessment, and plan effective surveillance, early response, and mitigation strategies.

The United States Department of Agriculture (USDA) has been working with state wildlife agencies to investigate the occurrence of SARS-CoV-2 across the range of WTD [16] and examine its evolutionary patterns [5]. National-scale surveillance data were collected by opportunistically sampling hunter-harvested deer and through targeted agency management. However, the epidemiological dynamics of SARS-CoV-2 emergence in WTD and ecological drivers of this emergence have not been studied closely. Estimates for epidemiological dynamics can guide risk assessments for infection emergence events and risk-based surveillance plans to study infection transmission rates, spread, and duration.

National surveillance data can reveal landscape-scale spatial variation in infection that may be linked to regional and environmental factors [17, 18]. Although individual outbreaks occur at local scales, variation between outbreaks can arise from complex interactions between environmental conditions and infection transmission rates [19]. Landscape-scale analyses routinely incorporate spatial statistical models to evaluate the consistency (i.e., predictability) of potential risk factors while accounting for the impact that geographic proximity (i.e., spatial correlation) can have on empirical patterns [20]. For example, spatial correlation can quantify the probability that neighboring local outbreaks may naturally co-occur, even in the absence of predictive environmental risk factors.

We embed spatially and temporally correlated epidemiological compartment models within a hierarchical statistical model to estimate the dynamics of concurrent outbreaks of SARS-CoV-2 in WTD across the conterminous United States (CONUS). The epidemiological models quantify spatially varying infection parameters, such as transmission rates. The statistical framework partitions uncertainty to account for the unbalanced spatial, temporal, geographic, and demographic distribution of samples that arises from opportunistic sampling (e.g., more male vs. female WTD sampled). Hierarchical modeling frameworks can identify epidemiological parameters that best explain empirical infection patterns [21–24]. Epidemiological compartment models are known to provide informative predictions for SARS-CoV-2 deaths in humans [25].

We use the hierarchical statistical model to study landscape-scale factors that influence the epidemiological dynamics of SARS-CoV-2 in WTD from national surveillance data that captures multiple outbreaks. We estimate demographic differences in infection, spatially varying epidemiological characteristics such as the effective reproductive ratio, and spatially varying estimates for the dates of peak infection. We also estimate potential spillover risk of infection from humans to WTD. The hierarchical model estimates ecological factors that can potentially explain the spatially varying differences. The model's spatial component makes it possible to predict emergence dynamics in areas where surveillance data have not been collected, to guide risk assessment and surveillance plans critical for One Health initiatives.

## 2. Methods

### 2.1. Data

#### 2.1.1. Surveillance of SARS-CoV-2 in White-Tailed Deer

We present a detailed epidemiological analysis of data collected from surveillance studies [16, 26]. Sampling for this surveillance program was opportunistic and did not follow a preset sampling design. Postmortem WTD samples were collected voluntarily from multiple sources, including hunter-harvest samples collected by state departments of natural resources, management events conducted by USDA Animal and Plant Health Inspection Service (USDA-APHIS), Wildlife Services, and sampling of miscellaneous mortalities such as roadkill collected by all agencies. Sample source and individual deer-specific metrics including sex and age class were recorded. Removal location data were collected at the county level. When available, hunters were asked to disclose the county of removal, but in lieu of removal county, the check station county from where the sample collected was used. Nasal or oral swabs were collected and tested for the presence of SARS-CoV-2 viral RNA via rRT-PCR [5, 16, 26].

#### 2.1.2. County-Level Covariates

We use the 2020 Census Bureau population data [27] to estimate the human density for each county (residents per sq. km.). We use the United States Geological Survey's Gap Analysis Project (GAP) WTD species distribution model [28] to calculate the proportion of each county's land that can support WTD populations (i.e., WTD habitat). The GAP model uses empirical analyses of occupancy by habitat to predict the species occurrence across landcover classes. GAP landcover class pixels are converted to a binary based on if that pixel represents suitable year-round WTD habitat. We used the total area covered by WTD habitat pixels within a county divided by the total county area to calculate the proportion of WTD habitat in each county.

#### 2.1.3. County-Level Time-Varying Mortality Rates for SARS-CoV-2 in Humans

We compare SARS-CoV-2 surveillance data for humans to the SARS-CoV-2 surveillance data for WTD to evaluate the potential frequency of spillover from humans to deer at landscape scales. The SARS-CoV-2 pandemic in humans is difficult to track precisely. Public health departments use case counts, hospital admissions, mortality data, and derived metrics such as the proportion of all weekly deaths attributable to SARS-CoV-2 to monitor the state of the SARS-CoV-2 pandemic in humans [29, 30]. Each metric is susceptible to over- and under-reporting biases, which motivates recommendations for using excess mortality to monitor the pandemic instead [31]. Excess mortality is typically defined as the difference between the number of predicted all-cause deaths and the number of observed all-cause deaths, with the difference being attributed to SARS-CoV-2 [32]. However, excess mortality can be challenging to use at local scales since it can be negative and sensitive to the risk that pandemic-related behavioral changes (i.e., driving less) biases all-cause death predictions to be high [32, 33].

We use the weekly death rate of SARS-CoV-2 in humans as a lagged proxy to quantify the relative amount of human SARS-CoV-2 infection. Human SARS-CoV-2 mortality can be predicted reasonably well, which suggests reporting biases for mortality rates may be consistent across time and space, especially as compared to case counts that strongly depend on testing rates [25]. We calculated the weekly death rate of SARS-CoV-2 in humans per county using data from The New York Times repository of SARS-CoV-2 cases (deaths per 100,000 people between Sunday and Saturday). The New York Times data aggregates daily case and death counts published by state and local health departments.

### 2.2. Statistical Analyses

#### 2.2.1. Spatially Varying SIR Model

We specify a hierarchical Bayesian model that uses sample-level test results to estimate epidemiological parameters, associations with potential risk factors, and prevalence over time. We estimate separate epidemiological parameters for each county, within which we assume there is a local, well-mixed population of WTD. Landscape-scale variation in infection arises from differences in parameters across counties.

Spatially and temporally correlated, county-level susceptible–infected recovered (SIR) compartmental models account for trends across time and space. The model uses both sample- and county-level covariates to influence SIR model parameters, identifying potential risk factors for infection transmission. We apply the model to 2,893 counties across CONUS estimated to support WTD populations and focus on the weeks over which samples were collected.

The model's response variable, Y_*k*_ encodes the binary rRT-PCR test results for the *k*^th^ sample such that Y_*k*_=1 for positive results and Y_*k*_=0 for negative results. The model treats Y_*k*_ as a Bernoulli random variable with probability p_*k*_ of being positive. We interpret p_*k*_ as the individual test positivity or prevalence of SARS-CoV-2 for the *k*^th^ animal's group, time, and location. The model uses the regression function specified via

(1) $$\begin{array}{c} \begin{array}{c} \text{logit}\left({\text{p}}_{k}\right)=\sum_{j}{\text{a}}_{j}{\text{z}}_{kj}+\text{logit}\left({\text{i}}_{\ell_{k}}\left({\text{t}}_{k}\right)\right), \end{array} \end{array}$$

to link rRT-PCR test results to county-level SIR curves and sample-level covariates and external conditions (e.g., age, sex, and human death rate). The a_*j*_ and z_*kj*_ terms specify sample-level coefficients and covariates that adjust the baseline infected compartment i_*ℓ*_*k*__(·) of the SIR curve for county *ℓ*_*k*_ at time t_*k*_ based on group-level characteristics and external conditions for sample *k*, respectively. Covariates include main effects and select pairwise interactions for animal age class and sex, harvest source, and swab type (see Table S1 for detailed covariate listing). We assume counties are small enough for local WTD populations to be well-mixed, so that sampled deer are representative of their respective, within-county demographic groups.

We use the SIR curve to model the proportion of susceptible s_*ℓ*_(*t*), infected i_*ℓ*_(*t*), and recovered r_*ℓ*_(*t*) individuals in county *ℓ* at time *t* via spatially and temporally correlated systems of differential equations. The SIR system of differential equations for each county specified via

(2) $$\begin{array}{c} \frac{d{\text{s}}_{\ell }\left(t\right)}{dt}=\beta_{\ell }{\text{i}}_{\ell }\left(t\right){\text{s}}_{\ell }\left(t\right),\frac{d{\text{i}}_{\ell }\left(t\right)}{dt}=\beta_{\ell }{\text{i}}_{\ell }\left(t\right){\text{s}}_{\ell }\left(t\right)-\gamma {\text{i}}_{\ell }\left(t\right),\frac{d{\text{r}}_{\ell }\left(t\right)}{dt}=\gamma {\text{i}}_{\ell }\left(t\right), \end{array}$$

uses a population-level recovery parameter *γ* and spatially varying deer-to-deer contact rate *β*_*ℓ*_. Each county's SIR curve is modeled with a local outbreak time *t*_0,*ℓ*_ and common initial conditions s_*ℓ*_(*t*_0,*ℓ*_)=s_0_^*∗*^, i_*ℓ*_(*t*_0,*ℓ*_)=i_0_^*∗*^, and r_*ℓ*_(*t*_0,*ℓ*_)=r_0_^*∗*^. The SIR model's infectious period assumptions induce exponential growth in population-level infection before fade out. Modeling SIR parameters and initial conditions with respect to spatial random effects and covariates accounts for spatial and temporal similarities in SIR curves between counties.

We model the county-level contact rate *β*_*ℓ*_ relative to the recovery rate *γ* scaled by a SARS-CoV-2 local effective reproduction ratio R_*ℓ*_ for each county, such that *β*_*ℓ*_=*γ*R_*ℓ*_. The local effective reproduction ratio quantifies the number of WTD to which a single infected WTD can be expected to transmit SARS-CoV-2 to naïve contacts. Covariates and spatially correlated random effects influence R_*ℓ*_ via

(3) $$\begin{array}{c} \begin{array}{c} g\left({\text{R}}_{\ell }\right)=\sum_{j}{\text{b}}_{j}{\text{x}}_{\ell j}+\eta_{\ell }, \end{array} \end{array}$$

to link R_*ℓ*_ to county-level covariates that can influence deer-to-deer contact rates (e.g., habitable area and human population density). The link function *g*(·) is an exponentially smoothed ramp that is linear for 0.1 < R_*ℓ*_ < 10 and decays to a low of R_*ℓ*_=0 and a high of R_*ℓ*_=15 (additional details in Supplementary Materials). The b_*j*_ and x_*ℓj*_ terms specify county-level effects and covariates, and *η*_*ℓ*_ specifies a spatially correlated random effect for each county (see Table S1 for detailed covariate listing). A conditional autoregressive (CAR) process model uses county adjacency reference information to model spatial connection and correlation for *η*_*ℓ*_ [34]. The CAR model requires a spatial precision parameter *τ*_*ℓ*_ and a spatial range parameter *γ*_*ℓ*_, both of which are estimated from data. We also use a CAR process to model the local outbreak time *t*_0,*ℓ*_. Like *η*_*ℓ*_, the CAR model for *t*_0,*ℓ*_ requires a spatial precision parameter *τ*_*t*_0__ and spatial range parameter *γ*_*t*_0__. In conjunction with the other SIR curve parameters, the local outbreak time *t*_0,*ℓ*_ influences the time at which peak prevalence occurs.

We use Markov chain Monte Carlo (MCMC) methods to fit the model. MCMC procedures and prior distributions are described in the Supplementary Materials (Table S2).

#### 2.2.2. Spatio-Temporal Risk Evaluation and Mapping

The SIR model Equation (2) can estimate spatially and temporally complete maps of SARS-CoV-2 prevalence for WTD after model fitting, filling in data collection gaps. Model fitting estimates SIR parameters for all counties *ℓ* and times *t*; so, it is possible to estimate baseline prevalence i_*ℓ*_(*t*) and other compartments at any point in time and space. Model fitting also estimates sample-level coefficients a_*j*_; so, it is also possible to replace the variables z_*kj*_, *ℓ*_*k*_, and *t*_*k*_ in Equation (1) with appropriate substitutions z_*Gj*_, *ℓ*, and *t* to estimate prevalence p_*Gℓt*_ for an arbitrary demographic group and sample type *G* in county *ℓ* and time *t*. Within the Bayesian framework, composition sampling is the technical method that propagates uncertainty and dependance from estimates of parameters to estimates of prevalence, maps, and other features [34]. The prevalence p_*Gℓt*_ can be aggregated across both time and space, independently or together.

The time-averaged prevalence p_*Gℓ*_ for demographic group and sample type *G* in county *ℓ* is the average of the weekly prevalences p_*Gℓ*1_, p_*Gℓ*2_, p_*Gℓ*3_,…. Maps of p_*Gℓ*_ can illustrate where infection tended to be more widespread across the study period. Time-averaged prevalence also provides a metric that can be compared to empirical studies that present summary statistics of raw surveillance data. Composition sampling, again, propagates uncertainty and dependance from estimates of parameters to estimates of p_*Gℓ*_.

The space-averaged prevalence p_*GAt*_ for demographic group and sample type *G* in area *A* summarizes all prevalence estimates p_*Gℓt*_ for *G* at time *t* in area *A*. The summary p_*GAt*_ is a flexible weighted average specified via

(4) $$\begin{array}{c} \begin{array}{c} {\text{p}}_{GAt}=\sum_{\ell }{\text{w}}_{A\ell }{\text{p}}_{G\ell t}, \end{array} \end{array}$$

where w_*Aℓ*_ is the relative weight (or contribution) of county *ℓ* to area *A* at time *t*. For example, we can use Equation (4) to estimate overall prevalence in state *A* at time *t* by setting w_*Aℓ*_=0 for all counties outside state *A*. Within state *A*, we can set w_*Aℓ*_ proportional to the total area of state A's WTD habitat that falls within county *ℓ*. So, if 20% of state *A*'s WTD habitat falls within county *ℓ*, then we set w_*Aℓ*_=.2. As with p_*Gℓ*_, composition sampling propagates uncertainty and dependance from estimates of parameters to estimates of p_*GAt*_.

#### 2.2.3. Spillover Risk

We compare prevalence estimates that are both space and time-averaged to evaluate spillover. We use conditional probabilities to quantify spillover as the risk that, on average, an infected deer was infected due to human infection pressure. Using aggregation methods described previously, the sample-level model Equation (1) can estimate p_*DH*_, the time-averaged proportion of deer that were infected with SARS-CoV-2 across CONUS. The sample-level model can also estimate p_*D*_, the time-averaged proportion of deer that were infected with SARS-CoV-2 across CONUS in the absence of human infection pressure (i.e., through deer-to-deer transmission and other zoonoses). The estimate for p_*D*_ uses the fitted model to predict prevalence with all human SARS-CoV-2 dataset to 0. The sample-level model is not designed to directly estimate the time-averaged proportion of deer infected due to human infection pressure p_*H*_, but we assume the causes of infection are mutually exclusive, implying p_*DH*_=p_*D*_+p_*H*_. The conditional probability p_*H*|*DH*_=1 − p_*D*_/p_*DH*_ exactly quantifies spillover as we defined it earlier. Composition sampling propagates uncertainty and dependance from estimates of parameters to estimates of p_*H*|*DH*_.

## 3. Results

### 3.1. Sample Composition and Descriptive Statistics

From October 2021 through March 2022 there were 10,217 nasal or oral swab samples from WTD tested from 27 states and Washington, DC. SARS-CoV-2 viral RNA was detected in 13% (1,307) of the 10,217 samples [16, 26]. The raw, apparent prevalence summaries are descriptive statistics that do not account for the opportunistic sample collection. There were similar numbers of samples collected from both sexes (males = 5,076 and females = 5,141), but SARS-CoV-2 viral RNA was detected more often in males (15%) relative to females (11%). Adults (8,000 samples) were more heavily sampled than juveniles (2,217 samples), but detection rates were similar in both groups (13% vs. 12%). Nasal swabs (9,343 samples) were collected more often than oral swabs (364 samples), and 510 samples had missing data describing swab type. Infection rates (i.e., proportion positive) appeared higher in oral and unknown swabs (16% and 17%, respectively) relative to nasal swabs (12%). For sample source, hunter-harvest samples were the most common (4,577 samples with 17% positive), followed by samples collected from USDA removal and management purposes (agency management; 3,866 samples with 11% positive), or other mortalities (e.g., roadkill; 1,774 samples with 6% positive). Hunter-harvest samples were collected during a shorter time window (i.e., hunting seasons), while agency management and other mortalities were collected more consistently throughout the full period of surveillance. Samples were collected from 589 of the 2,893 counties that WTD can inhabit in the conterminous United States (CONUS) [28], and samples were not necessarily collected at regular time intervals. Deer habitat is estimated via the Gap Analysis Project (GAP) species distribution model [28]. Here, we quantify deer habitat as the GAP-estimated proportion of a county's land area that is inhabitable to WTD.

### 3.2. Risk Factors

#### 3.2.1. The Model Can Estimate Population-Level Epidemic Characteristics of SARS-CoV-2 Outbreaks in WTD

We inferred the effects of ecological risk factors using a hierarchical model of the surveillance data that included a sample-level component for inferring test positivity probability p_*k*_ for each individual *k*=1,…, 10, 217. The SIR component of the model simultaneously estimates a local effective reproduction ratio R_*ℓ*_ for each county *ℓ*=1,…, 2, 893 that WTD can inhabit in CONUS. A calibration curve assesses model fit, validating that p_*k*_ predicted positive and negative test outcomes well (Figure S1), and that estimates of p_*k*_ are close to apparent prevalence (observed data) with underprediction in regions with high predicted prevalence. The model fit indicates the method can use landscape characteristics and spatial correlation between observed outbreaks to estimate plausible ranges for prevalence in more than the 589 counties from which samples were collected. The model fit indicates the method can also estimate epidemiological characteristics of SARS-CoV-2 in WTD, such as the timing of outbreaks and peak prevalence across counties.

#### 3.2.2. Sex and Sample Source Are Significant Sample-Level Variables

**Figure 1:**
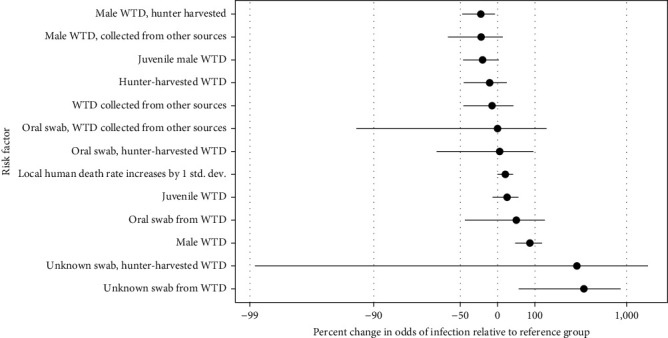
Estimated effects of logistic regression covariates on odds of infection relative to reference group (i.e., risk factors, *a*_*j*_ terms in Equation (1)). The reference group is oral swab samples from adult female WTD harvested by agency management.

We estimate that sample-level test positivity for agency harvested male WTD significantly increases relative to agency-harvested female WTD (Figures 1 and S2, additional details in Table S1; 14% positive males and 10% positive females from October 2021 through March 2022). The effect is moderated for hunter-harvested male WTD (10% positive males and 8% positive females from October 2021 through March 2022). We also estimate that test positivity is almost significantly decreased for juvenile male WTD. The surveillance data do not provide evidence that oral vs. nasal swab type or the main effect for age class (vs. the sex interaction) significantly impacts test positivity.

#### 3.2.3. Inhabitable Deer Area Effect Is Weaker than Human Population Density across Landscapes

For county-level effects, there are positive, but insignificant trends between the local effective reproduction ratio R_*ℓ*_ and covariates. The effects of deer habitat (a proxy for deer abundance) and human population indicate an insignificant, noisy positive trend (Figure S3, Rows b_2_ and b_3_ in Table S1). Predicted prevalence across counties in WTD increased from a posterior average of 10% when human population density was 10 people per sq. km. to 15% when human population density was 100 people per sq. km. from October 2021 through March 2022 (Figure S3). Predicted prevalence in WTD also increased from an average of 10% when the proportion of WTD habitat is low (i.e., near 0) to 15% when WTD habitat is high (i.e., near 1; Figure S3). Both potential trends are of biological interest, but are statistically insignificant due to substantial variation across counties.

#### 3.2.4. Human SARS-CoV-2 Infection Tends to Increase WTD SARS-CoV-2 Prevalence

The model estimates that SARS-CoV-2 prevalence in WTD tends to increase with SARS-CoV-2 infection in humans. The model estimates the odds of WTD prevalence increases by 13% for every additional 11 human deaths per 100,000 county residents (logistic regression parameter interpretation for row a_8_ in Table S1; 95% highest posterior density interval (HPDI) spans from 1% decrease to 31% increase). The model also estimates that, on average, 10% of positive deer detected were due to human infection pressure from October 2021 through March 2022 (95% HPDI: 0%–18%).

#### 3.2.5. Local Effective Reproduction Ratios Greater than 1 Are Widespread

**Figure 2:**
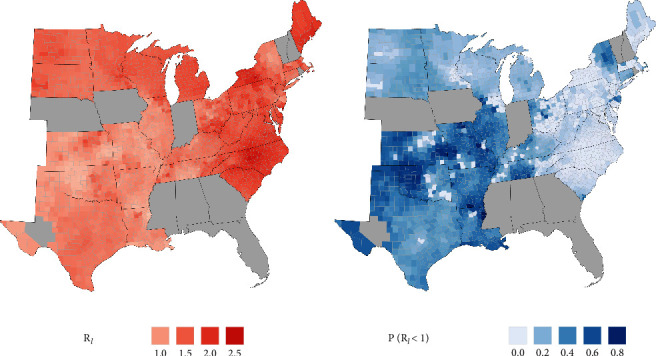
(a) Estimates for local effective reproduction ratio R_*ℓ*_ and (b) uncertainty (posterior probability that R_*ℓ*_ < 1). States that did not participate in the study are grayed out. Counties estimated through the GAP WTD species distribution model to not support WTD populations are also grayed out.

Estimates of the local effective reproduction ratio R_*ℓ*_ were greater than 1 in nearly all counties in states where samples were collected and ranged up to 2.5 in some counties (Figure 2(a)). However, there is also large uncertainty in R_*ℓ*_ estimates in states where few samples were collected such that R_*ℓ*_ could have been less than one for many mid- and south-western counties (Figure 2).

#### 3.2.6. Estimates of Time-Averaged Prevalence Were At Least 3% in Most Sampled Counties

**Figure 3:**
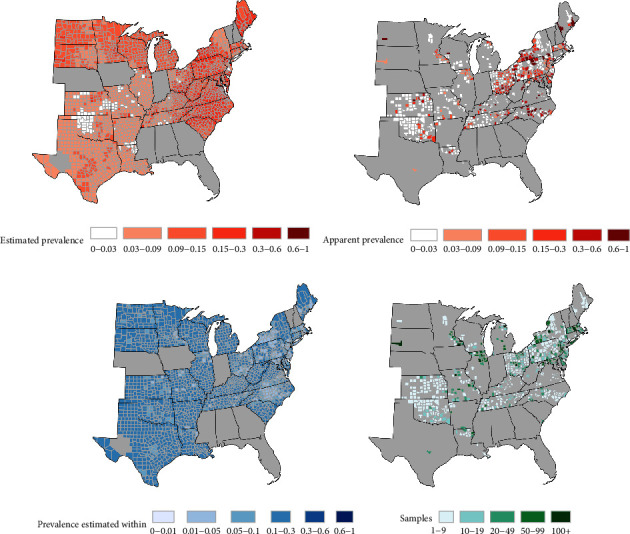
(a) Estimates for time-averaged prevalence from October 2021 through March 2022, (b) apparent prevalence from October 2021 through March 2022, (c) uncertainty for estimated prevalence (maximum half-width of 95% highest posterior density interval), and (d) number of samples collected from each county. Gray shading is as described for Figure 2.

Estimates of average prevalence from October 2021 through March 2022 tended to be higher on the east coast than in the mid- and south–west (i.e., time-averaged prevalence; Figure 3(a)). The model-based estimates adjust for uneven sample collection rates over time. The average county-level apparent prevalence (Figure 3(b); the proportion of positive test results per county) was more extreme (i.e., higher or lower) than time-averaged estimates in counties with low sample sizes (Figure 3(d)). Importantly, uncertainty in time-averaged prevalence estimates (Figure 3(c)) was also higher in counties with low sample sizes. Predicted peak prevalence varied spatially across the range of WTD studied.

#### 3.2.7. Peak Prevalence Occurred Earliest in Counties in the Northeast and Mid-Atlantic

**Figure 4:**
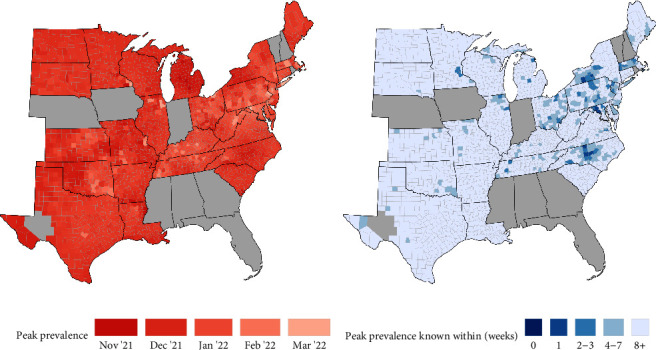
(a) Estimates for peak prevalence time with (b) uncertainty (maximum half-width of 95% highest posterior density interval). Gray shading is as described for Figure 2.

**Figure 5:**
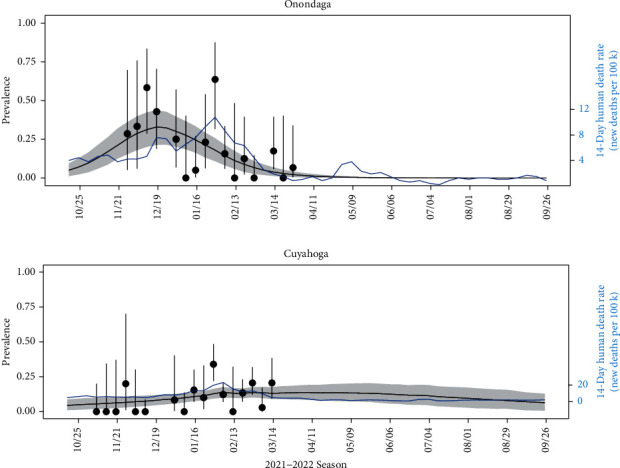
Estimated prevalence (solid black line) with uncertainty (95% HPD interval as gray shading) in the two most intensively sampled counties, (a) Onondaga county, New York (252 samples) and (b) Cuyahoga county, Ohio (609 samples). Blue time series shows the human death rate for both counties during the same time period. Black dots depict apparent prevalence (i.e., sample proportion of positive tests), with error bars from 95% frequentist intervals for proportions.

Peak prevalence occurred later in counties in the midwest and southeast (Figure 4(a)). However, there was local variation across counties within a state. In New York, peak prevalence is predicted to have occurred 1–3 months earlier in the western counties compared to the eastern counties (Figure 4(a)). However, uncertainty in predicted timing is higher in the eastern counties of New York compared to the western counties (Figure 4(b)). Examination of SARS-CoV-2 prevalence in WTD over time predicted outbreak start, peak prevalence, and prevalence decline occurred earlier in Onondaga County, New York than in Cuyahoga County, Ohio; the two most intensively sampled counties in our study (Figure 5). Comparison to human death rate data illustrates how SARS-CoV-2 in humans is not necessarily a primary driver for SARS-CoV-2 prevalence in WTD, but can prolong the duration of an outbreak in WTD.

## 4. Discussion

We identify ecological drivers of spatially varying outbreak dynamics and infer outbreak sizes, timing, and epidemiological parameters across the full range of WTD. Outbreaks were well characterized by assuming a single epidemic peak followed by fade out. We estimated that the R_*ℓ*_ (i.e., locally varying R_0_) ranged between 1 and 2.5, and that infection trends in humans may have contributed to 10% of infections in WTD. Evaluation of ongoing monitoring data will help evaluate persistence and whether multiple-peak epidemic models would better describe the infection process over longer time scales. Our methods provide landscape-scale surveillance programs a framework to infer population-level epidemiological processes from nonrandom sampling designs.

We provide an approach for estimating population-level outbreak parameters from multiple, sparsely observed outbreaks. Model-based analyses of surveillance data estimate infection prevalence at all points in space and time to fill in data collection gaps. Prevalence estimates can be interpreted as reconstructions of infection trajectories. Spatially analyzing reconstructed infection trajectories can identify regions that have been heavily impacted by infection and are potentially at increased risk for future outbreaks.

Our model estimates that SARS-CoV-2 in humans explained a substantial proportion of prevalence in WTD (10%) in the initial outbreaks. The result suggests human-to-deer spillover rates were high, are potentially important for persistence, and may be useful for informing targeted, risk-based surveillance. Phylogenetic studies corroborate our finding through the identification of many cases of human-to-deer transmission. However, the sampling design of these studies has prevented them from estimating population-level spillover rates [2, 3, 5, 26]. While SIR models do not identify individual spillover events, the human infection proxy within the sample-level model Equation (1) estimates the relative frequency of deer-to-deer vs. human-to-deer transmission events. In general, spillover can occur through direct contact between animals, or indirectly through excretions, blood, or intermediate hosts [35, 36]. Targeted surveillance programs that closely monitor small groups of wild animals are important for identifying likely pathways for spillover of SARS-CoV-2 from humans to WTD. Future studies with finer-scale data may also attempt to use a two-host system to closely model and quantify the impact of spillback from deer to humans on disease transmission and persistence [37].

Interpretation of epidemiological parameters, such as R_*ℓ*_, inherently depends on the specified disease model and its assumptions. Our model fits apparent prevalence well, with some underprediction in areas of high apparent prevalence. Improved sampling might improve model fit by reducing the effect of potential sampling bias on model fit diagnostics, or by better resolving potential risk factors and temporal trends. Disease models with more flexible assumptions about infectious periods, such as those that more closely model latent infectious periods [38], will inherently yield different reproductive ratios that could potentially better describe epidemiological dynamics if model fit is improved. However, waning immunity and changing demographics may be more appropriate extensions to the basic SIR modeling presented. But, such models require more precise demographic data and longer surveillance than are available.

An understanding of risk factors that drive epidemiological dynamics can be leveraged to predict potential patterns in future outbreaks. Evidence for substantial population-level spillover risk suggests that focusing surveillance of WTD in regions near human SARS-CoV-2 outbreaks would lead to finding the most samples that are positive for SARS-CoV-2. However, it is currently unclear if humans are infecting WTD close or far from their place of residence. Additional surveillance data could help obtain the best information for risk assessment for variants of concern in active circulation. Pathways for spillover can also be better assessed by collecting more data on deer-to-human interactions through camera studies and surveys that ask participants to describe their interactions with wildlife.

Posterior summaries for the risk factors identified in Figure 1 suggest potential strategies to optimize SARS-CoV-2 monitoring in future surveillance, with additional details in Table S1. Surveillance plans must balance resources between studying transmission and persistence to improve risk, assessment, and managing infection through control [39]. Descriptive summaries of the raw data suggested that prevalence differed for sample source (i.e., hunter vs. agency) and swab type (i.e., oral vs. nasal). However, the model did not find strong evidence for this pattern once the imbalanced sampling design factors were accounted for together. So, surveillance data collected from different sources and methods can likely be analyzed together without concern, similar to some rabies surveillance data [40]. The model also suggests male deer were infected at higher rates than female deer, implying that sampling male deer can increase chances of detecting SARS-CoV-2 in WTD populations when surveillance resources are limited. Sex-linked differences have also been identified through other surveillance programs [2, 4, 16, 26].

Local effective reproductive ratio of SARS-CoV-2 in WTD appeared to weakly increase with human population density. This might suggest that areas with higher human density have greater opportunity for zoonotic transmission, contributing to the force of infection in deer. Regional studies have also identified different infection rates with respect to broader, urban vs. rural land designations [26]. The effect of human density was relatively small with ample variation. Our model did not consider changes to human density across time, which likely does not accurately reflect human movement and contact patterns with deer because we did not have such data. For instance, the effect of areas such as campgrounds that see pulses of human density at irregular time intervals (i.e., around holidays) would not be captured by static landscape covariates [40]. Furthermore, natural areas such as parks and campgrounds that have pulses of human activity are also places where humans are likely to encounter a deer. Finer scale data on human mobility and human–deer contact frequencies in different settings would improve our understanding of this relationship and enable identification of additional landscape variables that could help identify how spillover is occurring and be included in risk mapping.

The model also suggested the local effective reproductive ratio increased with the proportion of a county's land that supports WTD populations, albeit weakly. Surveillance programs may choose to prioritize sampling counties with ample WTD habitat, which are also assumed to be counties with larger WTD populations. In lieu of using WTD density estimates, we used the proportion of a county's land that WTD can inhabit (i.e., WTD habitat) to approximate where WTD might be more densely populated. We chose this approach because WTD density information is limited to small-scale studies due to the difficulty of collecting this data [41], and methods for state-level abundance estimation vary across states, which introduces additional variation. Increased habitat suitability is tied to increased incidences of CWD in WTD [42], with the supporting hypothesis that suitable habitat supports higher density of WTD. The effect seen here might suggest infection reproduction is facilitated through deer-to-deer contact. However, finer scale WTD density information or habitat data that more closely informs WTD density would provide further insight to this relationship.

Infection transmission pressure from humans to deer is difficult to quantify because reporting rates in humans can vary widely, making infection surveillance in humans challenging, but our method suggests proxies (i.e., human death rate) can be effective tools for surveillance of SARS-CoV-2 in WTD. However, the proxy has likely become increasingly uninformative (after the time frame of this study) as effective treatments and vaccination have become available and survival has increased, even when infection rates are high. Future evaluation of SARS-CoV-2 in WTD may require different proxies for human infection. Surveillance of SARS-CoV-2 in humans requires extensive funding and consistent community participation, and is further challenging because positive at-home tests are generally not included in official reporting. Public health priorities also impact the availability of human SARS-CoV-2 surveillance data [30]. One Health approaches toward disease surveillance can potentially help provide structure to improve sampling efforts across species. Long-term monitoring can also provide data to evaluate predictive models.

Quantifying infection dynamics requires intensive data distributed throughout time and space. In this study, we used an opportunistic sampling design, which incurred temporal and spatial data gaps. Model-based analyses accounted for uneven sampling and estimate infection dynamics between data collection gaps. The model propagates uncertainty in our estimates of SARS-CoV-2 prevalence in WTD (Figure 3(c)), and uncertainty in these estimates could be reduced through continued sampling in counties where long-term sampling has already taken place. Furthermore, new sampling in counties that do not currently have data and are distant from well-sampled counties (e.g., represent different values in of covariates such as proportion of land inhabitable to WTD, human density, human case rates, or other potential risk factors that have yet to be explored) would bolster the confidence of these estimates. However, requirements for reducing estimate uncertainty can change over time, and would be best addressed using an adaptive sampling design. Future surveillance programs may also reduce uncertainty in county-level estimates by intensively sampling individual WTD populations within a subset of counties where samples are collected. Sampling individual WTD populations within counties can augment landscape-scale data through expanded hierarchical models, improving estimates of transmission dynamics and their risk factors. Similarly, uncertainty can also be reduced via repeated, long-term sampling at specific locations spread across different ecosystems, focusing both on humans and WTD. Such sampling can help to disentangle the drivers of infection dynamics and persistence both within and across populations—the subject of our ongoing work.

## 5. Conclusions

Estimates of outbreak parameters and their corresponding risk factors can help optimize strategies for risk-based surveillance, prevention, early response, and control of zoonotic diseases. Optimization is important because surveillance programs can only partially observe disease trajectories due to limited resources. Our work demonstrates how prevalence estimates can be interpreted as reconstructions of disease trajectories. Combining estimates of prevalence across points in space and time helps to fill data collection gaps for population-scale inference of epidemiological parameters that can be used to understand drivers of transmission risk and disease hotspots in a newly emerging disease at the human–animal interface.

## Data Availability

The complete dataset analyzed in this study is not publicly available due to sensitive sample-level collection information, such as detailed sample collection locations and dates, but can potentially be made available from the corresponding author on reasonable request. Key information about sample sizes and model output, such as fitted surfaces, are provided as supplementary materials at https://doi.org/10.15482/USDA.ADC/24926433.

## Supplementary Materials

Supplementary Materials The supporting information file provides additional details about the model parameters, prior distribution, posterior distribution and estimates, and model fit. It also contains supplementary Tables S1 and S2, and Figures S1–S4.
