# Supplementary material for: Landscape-Scale Epidemiological Dynamics of SARS-CoV-2 in White-Tailed Deer
Source: Transbound Emerg Dis. 2024 Feb 10;2024:7589509. doi: 10.1155/2024/7589509 (PMC12017121; doi:10.1155/2024/7589509)
Supplement: Supplementary Materials — The supporting information file provides additional details about the model parameters, prior distribution, posterior distribution and estimates, and model fit. It also contains supplementary Tables S1 and S2, and Figures S1–S4. [file 7589509.f1.pdf]

Supporting information for Epidemiological Dynamics of SARS-CoV-2 in White-tailed Deer

Joshua Hewitt*[6], Grete Wilson-Henjum[6], Derek T. Collins[2], Timothy J. Linder[2], Julianna B. Lenoch[2], Jonathon D. Heale[3], Christopher A. Quintanal[4], Robert Pleszewski[4], Dillon S. McBride[5], Andrew S. Bowman[5], Jeffrey C. Chandler[4], Susan A. Shriner[1], Sarah N. Bevins[2], Dennis J. Kohler[2], Richard B. Chipman[3], Allen L. Gosser[3], David L. Bergman[3], Thomas J. DeLiberto[3], Kim M. Pepin[1]

[1]National Wildlife Research Center, Wildlife Services, Animal and Plant Health Inspection Service, United States Department of Agriculture, Fort Collins, CO, USA
[2]National Wildlife Disease Program, Wildlife Services, Animal and Plant Health Inspection Service, United States Department of Agriculture, Fort Collins, CO, USA
[3]Wildlife Services, Animal and Plant Health Inspection Service, United States Department of Agriculture, Fort Collins, CO, USA
[4]Wildlife Disease Diagnostic Laboratory, Wildlife Services, Animal and Plant Health Inspection Service, United States Department of Agriculture, Fort Collins, CO, USA
[5]Veterinary Preventive Medicine, The Ohio State University College of Veterinary Medicine, Columbus, OH, USA
[6]Department of Wildland Resources, Utah State University, Logan, UT, USA

1 Supplemental results

A calibration curve showed that p_k predicted positive and negative test outcomes well (Figure S1). We visualize differences between posterior distributions of time-averaged prevalence across demographic groups and management types in Figure S2. The net impact of covariates is visualized by plotting estimated prevalence across the covariates (Figure S3). Parameter estimates for the model are presented in Table S1.

2 Exponentially smoothed ramp

We design a bounded, piecewise continuous function $g^{-1} : \mathbb{R} \to (L, U)$ that is linear over the subdomain $[-r, r]$ and whose value exponentially decays to lower and upper asymptotes L and U, respectively. The implied function g provides an alternative link function to scaled and shifted logistic or probit transformations (Figure S4). Logistic and probit functions are common bounded transformation functions for statistical models but provide a limited range over which the transformations are approximately linear.

*Address correspondence to josh.hewitt@usu.edu

We specify the function g^{-1} via

$$g^{-1}(x;\lambda_1,\lambda_2,y_1,y_2,L,U,r) = \begin{cases} h(x;\lambda_1,-r,y_1,L,-1) & x \in (-\infty,-r) \\ \frac{y_2-y_1}{2r}(x+r)+y_1 & x \in [-r,r] \\ h(x;\lambda_2,r,y_2,U,1) & x \in (r,\infty) \end{cases}, \tag{1}$$

where $h(x;\lambda,x^*,y^*,A,\delta) = (y^*-A)e^{\delta\lambda(x^*-x)} + A$. The parameters L and U specify the function's lower and upper asymptotes, respectively. The parameter r defines the subdomain around the origin over which g^{-1} is linear. The values y_1 and y_2 define the endpoints $g^{-1}(-r) = y_1$ and $g^{-1}(r) = y_2$, which determine the linear slope. The scale parameters λ_1 and λ_2 can be tuned via numerical optimization to ensure the slope for g^{-1} is continuous for all $x \in \mathbb{R}$.

3 Model fitting procedures

All continuous covariates are scaled and shifted to have mean 0 and unit variance before model fitting. Standard prior distributions are assigned to unknown parameters (Table S2). Markov chain Monte Carlo (MCMC) methods are used to draw inference on model parameters and random effects. The model is run for 1,000,000 iterations, thinning to retain 10,000 samples to approximate the joint posterior distribution. Most of the spatial random effects for each county that support WTD populations across CONUS will not be precisely estimated because data is only collected from 589 of the 2,893 modeled counties. However, the model includes spatial random effects for all counties to inform spatial relationships between counties, and support risk mapping.

4 Prior distributions

Prior distributions are presented in Table S2. The distributions are parameterized such that Normal(μ,σ^2) has mean μ and variance σ^2, Inv-Gamma(a,b) has mean $b/(a-1)$ for $a > 1$ and variance $b^2/(a-1)^2/(a-2)$ for $a > 2$, Uniform(a,b) has mean $(a+b)/2$ and variance $(b-a)^2/12$, and Gamma(a,b) has mean a/b and variance a/b^2.

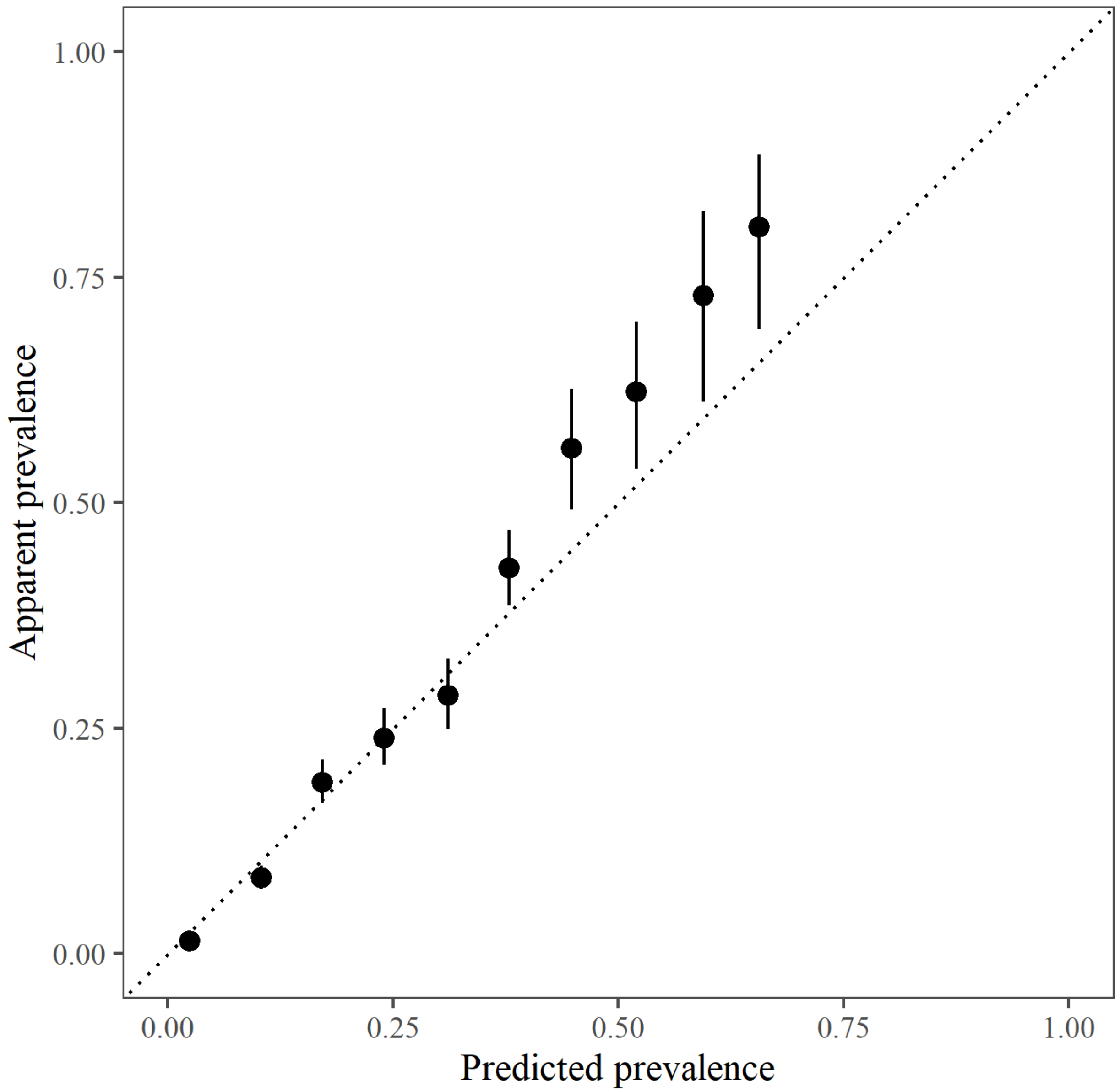

Figure S1: Calibration curve, comparing in-sample predictions of infection risk p_k (i.e., predicted prevalence) to observed outcomes against a 1:1 reference line (dotted line). Apparent prevalence (proportion of positive test results per group) is computed for model-fit diagnostic groups formed by binning predicted prevalence into 10 ranges. Error bars depict standard, frequentist 95% confidence intervals for each apparent prevalence group.

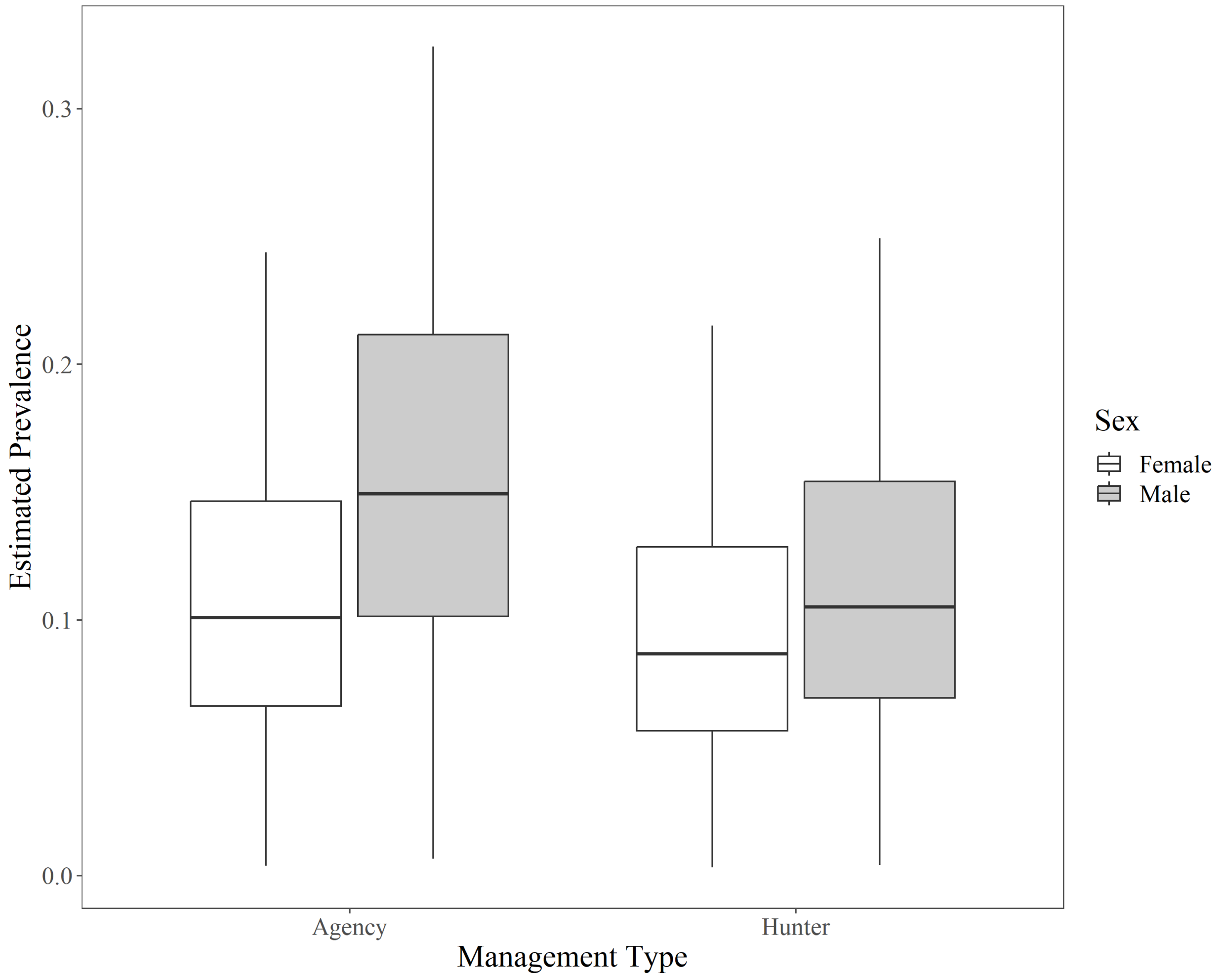

Figure S2: Distributions of posterior estimates for time-averaged prevalence across counties from October 2021 through March 2022 for different demographic groups and management types.

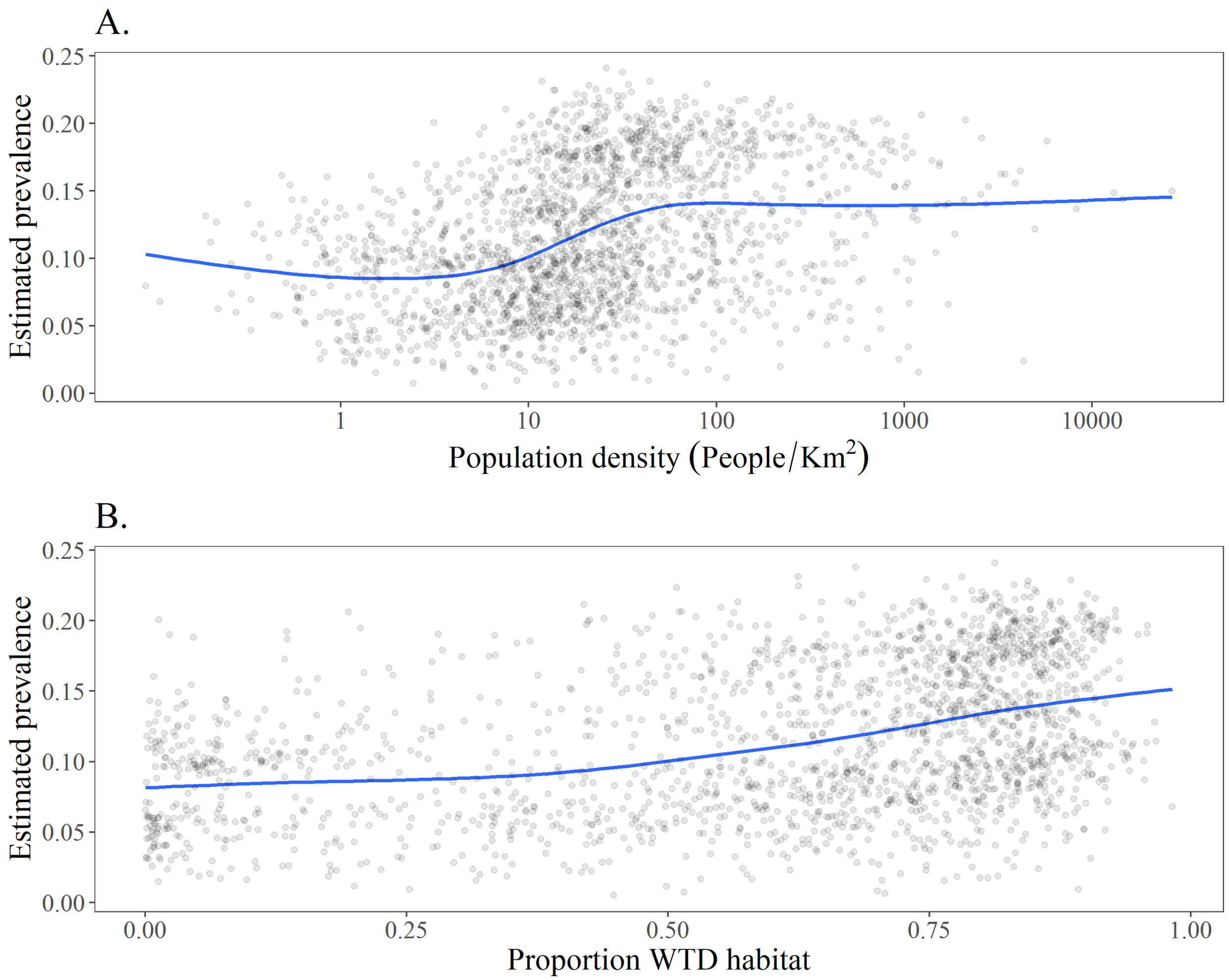

Figure S3: Estimates for time-averaged prevalence across counties plotted against ecological covariates from October 2021 through March 2022, with empirical trend line overlaid (blue; GAM smoother). Each point in the plot represents the time-averaged prevalence for one county.

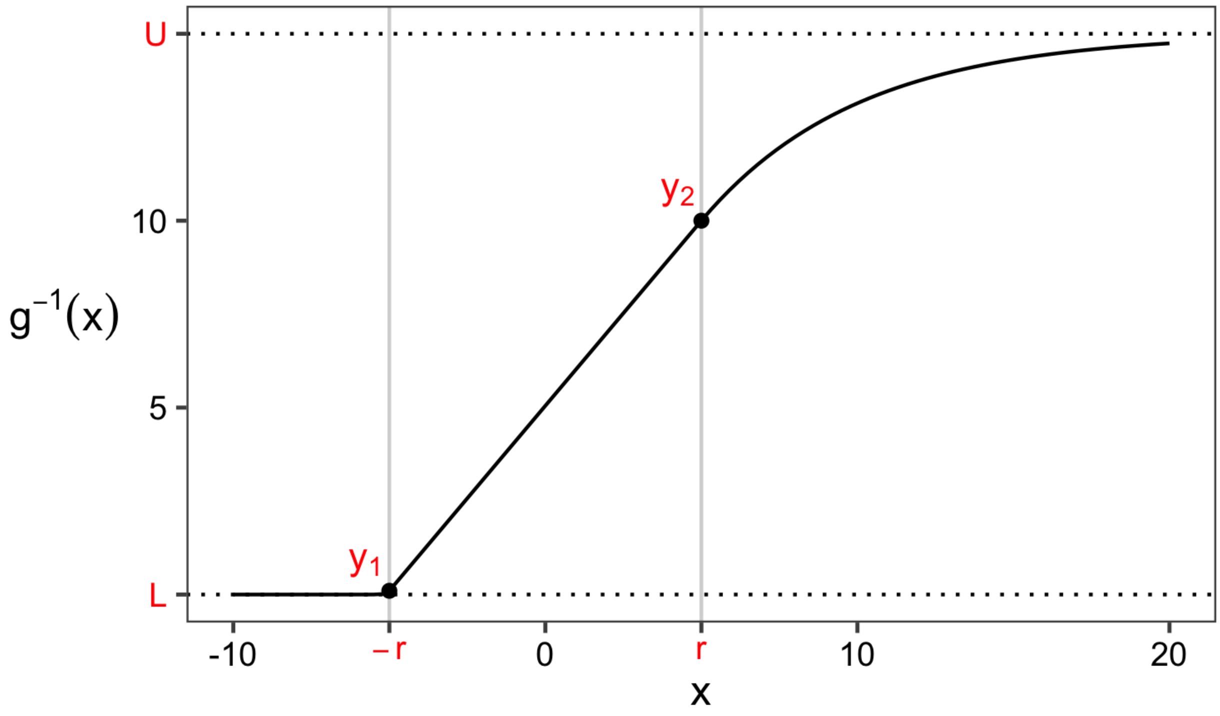

Figure S4: Exponentially smoothed ramp with $L = 0$, $U = 15$, $r = 5$, $y_1 = .1$, and $y_2 = 10$, and key parameters highlighted.

Table S1: Parameter descriptions and posterior distribution summaries for regression effects and spatial parameters. Posterior distribution summaries include the posterior mean, 95% highest posterior density interval (HPDI), and posterior standard deviation (s.d.).

**Component**	**Parameter**	**Covariate**	**Type**	**Post. mean**	**95% HPDI**	**Post. s.d.**
p_k	a_1	$z_{k1} = 1$	Intercept	1.95	(1.17, 2.69)	0.39
	a_2	$z_{k2} = (\text{Male})_k$	Group indicator	0.59	(0.35, 0.85)	0.13
	a_3	$z_{k3} = (\text{Juvenile})_k$	Group indicator	0.17	(−0.06, 0.41)	0.12
	a_4	$z_{k4} = (\text{Hunter harvest})_k$	Group indicator	−0.17	(−0.56, 0.22)	0.20
	a_5	$z_{k5} = (\text{Other management})_k$	Group indicator	−0.13	(−0.57, 0.34)	0.23
	a_6	$z_{k6} = (\text{Oral swab})_k$	Sample indicator	0.28	(−0.41, 0.98)	0.36
	a_7	$z_{k7} = (\text{Unknown swab})_k$	Sample indicator	1.50	(0.67, 2.40)	0.45
	a_8	$z_{k8} = (\text{Human death rate})_k$	Spillover pressure proxy	0.14	(0.00, 0.29)	0.07
	a_9	$z_{k9} = z_{k2}z_{k3}$	Interaction (Sex/Age)	−0.29	(−0.60, 0.05)	0.17
	a_{10}	$z_{k10} = z_{k2}z_{k4}$	Interaction (Sex/Management)	−0.32	(−0.61, −0.01)	0.15
	a_{11}	$z_{k11} = z_{k2}z_{k5}$	Interaction (Sex/Management)	−0.34	(−0.81, 0.16)	0.25
	a_{12}	$z_{k12} = z_{k4}z_{k6}$	Interaction (Management/Col'n.)	−0.05	(−0.87, 0.80)	0.43
	a_{13}	$z_{k13} = z_{k5}z_{k6}$	Interaction (Management/Col'n.)	−0.25	(−1.63, 1.15)	0.71
	a_{14}	$z_{k14} = z_{k4}z_{k7}$	Interaction (Management/Col'n.)	0.44	(−2.25, 3.42)	1.46
R_ℓ	b_1	$x_{\ell 1} = 1$	Intercept	−3.76	(−4.10, −3.32)	0.20
	b_2	$x_{\ell 2} = \log(\text{Human pop. density})_\ell$	County data	0.06	(−0.02, 0.16)	0.05
	b_3	$x_{\ell 3} = (\text{Prop. WTD habitat})_\ell$	County data	0.10	(−0.01, 0.24)	0.06
η_ℓ	τ_ℓ	N/A	Spatial precision	2.23	(0.51, 4.54)	1.12
	γ_ℓ	N/A	Spatial range	1.00	(0.99, 1.00)	0.00
$t_{0,\ell}$	τ_{t_0}	N/A	Temporal precision	0.07	(0.04, 0.12)	0.02
	γ_{t_0}	N/A	Temporal range	1.00	(0.99, 1.00)	0.00
$r_\ell(t)$	γ	N/A	Recovery rate	0.86	(0.53, 1.18)	0.17

Table S2: Prior distributions for model parameters.

**Component**	**Parameter**	**Distribution**
p_k	a_1	$\text{Normal}(0, 100)$
	a_2	$\text{Normal}(0, 100)$
	a_3	$\text{Normal}(0, 100)$
	a_4	$\text{Normal}(0, 100)$
	a_5	$\text{Normal}(0, 100)$
	a_6	$\text{Normal}(0, 100)$
	a_7	$\text{Normal}(0, 100)$
	a_8	$\text{Normal}(0, 100)$
	a_9	$\text{Normal}(0, 100)$
	a_{10}	$\text{Normal}(0, 100)$
	a_{11}	$\text{Normal}(0, 100)$
	a_{12}	$\text{Normal}(0, 100)$
	a_{13}	$\text{Normal}(0, 100)$
	a_{14}	$\text{Normal}(0, 100)$
R_ℓ	b_1	$\text{Normal}(0, 100)$
	b_2	$\text{Normal}(0, 100)$
	b_3	$\text{Normal}(0, 100)$
η_ℓ	τ_ℓ	$\text{Inv-Gamma}(2, 1)$
	γ_ℓ	$\text{Uniform}(-1.41, 1.00)$
$t_{0,\ell}$	τ_{t_0}	$\text{Inv-Gamma}(2, 1)$
	γ_{t_0}	$\text{Uniform}(-1.41, 1.00)$
$r_\ell(t)$	γ	$\text{Gamma}(2, 1)$
